# Impacts of triglyceride-glucose index on prognosis of patients with type 2 diabetes mellitus and non-ST-segment elevation acute coronary syndrome: results from an observational cohort study in China

**DOI:** 10.1186/s12933-020-01086-5

**Published:** 2020-07-08

**Authors:** Qi Zhao, Ting-Yu Zhang, Yu-Jing Cheng, Yue Ma, Ying-Kai Xu, Jia-Qi Yang, Yu-Jie Zhou

**Affiliations:** 1 Department of Cardiology, Beijing Anzhen Hospital, Capital Medical University, Beijing Institute of Heart Lung and Blood Vessel Disease, Beijing Key Laboratory of Precision Medicine of Coronary Atherosclerotic Disease, Clinical Center for Coronary Heart Disease, Capital Medical University, Beijing, 100029 China; 2 Research Center for Coronary Heart Disease, Fuwai Hospital, National Center for Cardiovascular Diseases, Chinese Academy of Medical Sciences and Peking Union Medical College, Beijing, 100037 China

**Keywords:** Triglyceride-glucose index, Type 2 diabetes mellitus, Non-ST-segment elevation acute coronary syndrome, Percutaneous coronary intervention

## Abstract

**Background:**

The relationship between triglyceride-glucose index (TyG index) and the prevalence and prognosis of cardiovascular disease has been confirmed by former studies. However, it remains uncertain whether TyG index has a prognostic impact in patients with type 2 diabetes mellitus (T2DM) and non-ST-segment elevation acute coronary syndrome (NSTE-ACS) undergoing percutaneous coronary intervention (PCI).

**Methods:**

The study retrospectively enrolled 798 patients (mean age: 60.9 ± 8.3 years; 68.3% men) with T2DM and NSTE-ACS who underwent PCI at Beijing Anzhen Hospital from January to December 2015. TyG index was calculated as previously reported: ln [fasting TGs (mg/dL) * FBG (mg/dL)/2]. The primary endpoint was a composite of adverse events as follows: all-cause death, non-fatal myocardial infarction (MI) and ischemia-driven revascularization.

**Results:**

TyG index was significantly higher in patients with a primary endpoint event compared with those without. Multivariate Cox proportional hazards analysis showed that 1-unit increase of TyG index was independently associated with higher risk of primary endpoint, independent of other risk factors [hazard ratio (HR) 3.208 per 1-unit increase, 95% confidence interval (CI) 2.400–4.289, P < 0.001]. The addition of TyG index to a baseline risk model had an incremental effect on the predictive value for adverse prognosis [AUC: baseline risk model, 0.800 vs. baseline risk model + TyG index, 0.856, P for comparison < 0.001; category-free net reclassification improvement (NRI) 0.346, P < 0.001; integrated discrimination improvement (IDI) 0.087, P < 0.001].

**Conclusions:**

Increased TyG index is a significant predictor of adverse prognosis in patients with T2DM and NSTE-ACS undergoing PCI. Further studies need to be performed to determine whether interventions for TyG index have a positive impact on improving clinical prognosis.

## Background

Coronary artery disease (CAD) has been recognized as the leading cause of disability and mortality in contemporary society. In recent years, in spite of superior evidence-based strategies including optimized drug therapy and revascularization having been widely developed and applied, the risk of recurrent adverse cardiovascular outcomes remains relatively high in patients with CAD, especially for those who have ever had an acute coronary syndrome (ACS) [1–3]. Previous studies have suggested that more than one-quarter of patients with ACS are combined with Type 2 Diabetes mellitus (T2DM), which has been widely proved to be one of the most significant risk factors for cardiovascular disease [4]. Certain studies have demonstrated that T2DM is significantly correlated with higher prevalence of CAD, more complex coronary lesions and worse prognosis [4–6]. Therefore, identification of the residual risk factors of diabetic patients with ACS is of great clinical importance if we are to develop new therapeutic targets and to tailor risk reduction strategies that match individual risk level.

Insulin resistance (IR), the critical mechanism of the pathogenesis of T2DM, has been extensively demonstrated to be significantly related to the development of coronary and carotid atherosclerosis and an increased risk of adverse prognosis [7–10]. The triglyceride-glucose index (TyG index), which is derived from fasting triglycerides (TGs) and fasting blood glucose (FBG), has been proposed as a surrogate biomarker of IR and former studies have proved that it has high correlation with hyperinsulinaemic-euglycaemic clamp (the gold standard technique for assessing IR), either in individuals with or without T2DM [11–13]. Studies have shown that an increased level of TyG index is closely related to higher incidence of diabetes and prediabetic status [14–16]. Furthermore, the association between TyG index and the prevalence and prognosis of cardiovascular disease has been confirmed by certain clinical researches, despite the existence of diabetes or not at baseline [17–22].

However, the prognostic significance of TyG index in patients with T2DM and non-ST-segment elevation acute coronary syndrome (NSTE-ACS) who were treated with percutaneous coronary intervention (PCI) has not been fully studied. Based on this, the present study was designed with the aim of: (1) identifying the potential association between IR quantified by TyG index and clinical prognosis; (2) determining whether TyG index has an incremental effect on risk stratification on the basis of traditional risk factors in participants with T2DM and NSTE-ACS undergoing PCI.

## Methods

### Study population

**Fig. 1:**
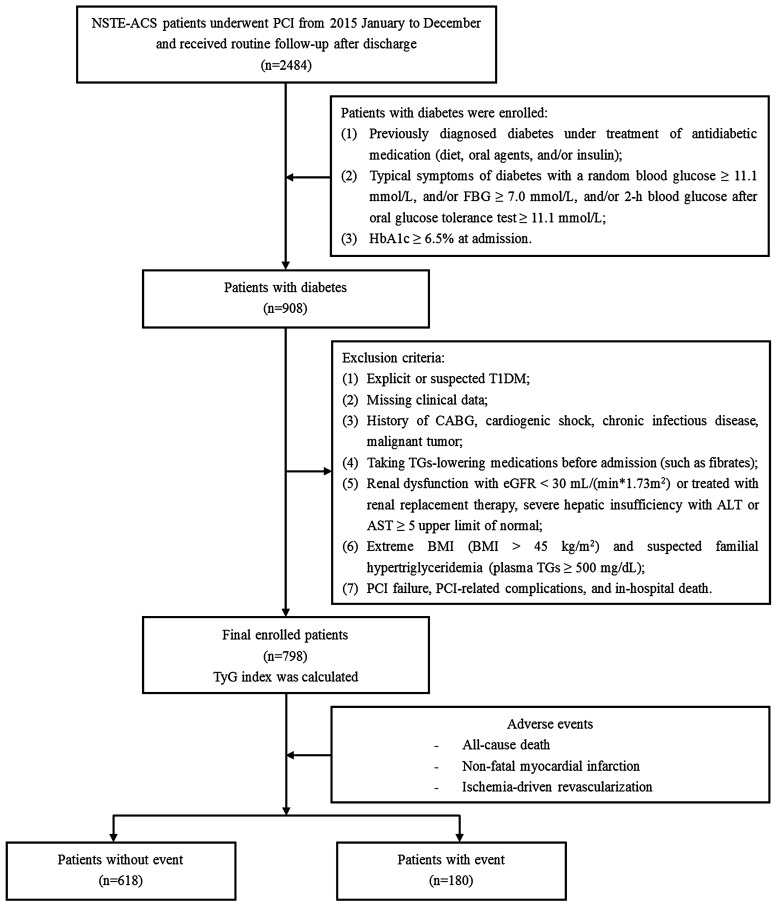
Flow chart of the study population enrollment. *NSTE-ACS* non-ST-segment elevation acute coronary syndrome, *PCI* percutaneous coronary intervention, *FBG* fasting blood glucose, *HbA1c* glycosylated hemoglobin A1c, *T1DM* type 1 diabetes mellitus, *CABG* coronary artery bypass grafting, *TGs* triglycerides, *eGFR* estimated glomerular filtration rate, *ALT* alanine transaminase, *AST* aspartate transaminase, *BMI* body mass index, *TyG* triglyceride glucose

The present study is a single-center, observational, retrospective cohort study among patients with diabetes who were diagnosed with NSTE-ACS and treated with elective PCI at Beijing Anzhen Hospital between January and December 2015. The exclusion criteria were listed as follows: (1) explicit or suspected type 1 diabetes mellitus (T1DM); (2) missing clinical data; (3) history of coronary artery bypass grafting (CABG), cardiogenic shock, chronic infectious disease and malignant tumor; (4) taking TGs-lowering medications before admission (such as fibrates); (5) extreme body mass index (BMI) (BMI > 45 kg/m^2^) and suspected familial hypertriglyceridemia [plasma TGs ≥ 500 mg/dL (5.65 mmol/L)]; (6) renal dysfunction with estimated glomerular filtration rate (eGFR) < 30 mL/(min * 1.73 m^2^) or treated with renal replacement therapy, severe hepatic insufficiency with alanine transaminase (ALT) or aspartate transaminase (AST) ≥ 5 upper limit of normal; (7) PCI failure, PCI-related complications, and in-hospital death. Ultimately, a cohort of 798 patients who met the enrollment principles were included for the present analyses (Fig. 1).

### Data collection and definitions

Data of demographic and clinical characteristics, including age, sex, weight, height, heart rate, blood pressure [systolic blood pressure (SBP) and diastolic blood pressure (DBP)], medical history, family history, and medical treatment were extracted from the medical information recording system of Beijing Anzhen Hospital. BMI was calculated as follows: BMI = weight (kg)/[height (m)]^2^. Criteria for diabetes include: (1) previously diagnosed diabetes under treatment of antidiabetic medication (diet, oral agents, and/or insulin); (2) the typical symptoms of diabetes with a random blood glucose ≥ 11.1 mmol/L, and/or FBG ≥ 7.0 mmol/L, and/or 2-h blood glucose after oral glucose tolerance test (OGTT) ≥ 11.1 mmol/L [23]; (3) glycosylated hemoglobin A1c (HbA1c) level ≥ 6.5% on admission [24]. NSTE-ACS was composed of non-ST-segment elevation myocardial infarction (NSTEMI) and unstable angina (UA), definitions of which were determined by appropriate guidelines [25]. NSTEMI was defined as having symptoms of ischemia and elevated cardiac troponin I (cTnI), and without an elevation of ST-segment. UA was diagnosed as ischemic symptoms at rest, or exacerbated or new-onset symptoms with transient ischemic ST-segment shifts, and without release of myocardial enzymes related to myocardial necrosis. Patients with SBP ≥ 140 mmHg and/or DBP ≥ 90 mmHg, or those receiving anti-hypertensive treatments were considered having hypertension. Peripheral vascular disease (PVD) was defined as aorta and other arteries than coronary arteries, with exercise related claudication, or reduced or absent pulsation, or angiographic stenosis of more than 50%.

Venous blood samples were collected after an overnight fasting on the day of the baseline coronary procedure. The routine hematology and biochemical parameters, including lipid profiles [TGs, total cholesterol (TC), low-density lipoprotein cholesterol (LDL-C), high-density lipoprotein cholesterol (HDL-C)], creatinine, uric acid, FBG, HbA1c, high-sensitivity C-reactive protein (hs-CRP), and other biomarkers, were determined by standard laboratory methods in central lab of Beijing Anzhen Hospital. Patients with fasting TC > 200 mg/dL, and/or LDL-C > 130 mg/dL, and/or TGs > 150 mg/dL, and/or HDL-C < 40 mg/dL at admission, and/or previously long-term use of lipid-lowering drugs were considered having dyslipidemia. The eGFR was calculated as previously described: eGFR [mL/(min * 1.73 m^2^)] = 186 * serum creatinine (mg/dL)^−1.154^ * age^−0.203^ (* 0.742 if female) [26]. Baseline TyG index was calculated based on fasting TGs and FBG values obtained at admission as previously reported: ln [fasting TGs (mg/dL) * FBG (mg/dL)/2] [11]. Left ventricular ejection fraction (LVEF) was evaluated by two-dimensional modified Simpson’s method using an ultrasonic cardiogram (Philips Company, Eindhoven, The Netherlands).

Coronary angiogram data were analyzed and recorded by at least two experienced cardiologists, and measurements of coronary artery lesion characteristics were obtained. The lesion characteristics were defined as follows: (1) multi-vessel lesion: more than two main coronary branches (vessel diameter ≥ 2 mm) with extent of stenosis ≥ 50%. (2) chronic total occlusion lesion: lesion with complete obstruction [thrombolysis in myocardial infarction (TIMI) flow grade 0] lasting longer than 3 months, which was judged from the previous medical history or coronary angiogram results. (3) diffuse lesion: a single stenotic lesion with a length of ≥ 20 mm. (4) bifurcation lesion: stenosis occurred adjacent to and/or involving the origin of a significant side branch that has too much functional value and so cannot be lost during the interventional procedure. (5) in-stent restenosis: stenosis of ≥ 50% occurring in the segment inside the stent, 5 mm proximal or distal to the stent [27]. The severity of coronary artery lesions was quantified by the synergy between PCI with taxus and cardiac surgery (SYNTAX) score. The SYNTAX score was calculated for each participant using the online calculator (http://www.syntaxscore.com). PCI was performed in accordance with current practice guidelines in China [28], and detailed strategies were determined by experienced interventional cardiologists.

### Follow-up and endpoint event

After baseline PCI, all patients were routinely followed up by trained professionals who were blinded to the baseline information at 3, 6, and 12 months and then annually for up to 36 months. The information about adverse prognostic events was obtained from patients or their family members by telephone questionnaire. The information was further confirmed by careful verification of corresponding medical records if necessary. The primary observational endpoint was defined as a composite of events including all-cause death, non-fatal myocardial infarction (MI) and ischemia-driven revascularization. The secondary observational endpoints were each component of the composite primary endpoint. MI was defined as elevated cardiac troponin higher than the upper reference limit with ischemia indicated from symptoms and/or electrocardiographic changes, with or without an elevation of ST-segment. Ischemia-driven revascularization was defined as the revascularization procedure associated with symptoms and/or electrocardiographic changes implicating ischemia. The first primary endpoint event that occurred during the follow-up was used for analysis in current study. For patients with multiple adverse outcomes occurring almost simultaneously during the follow-up, only the most severe event (all-cause death > non-fatal MI > ischemia-driven revascularization) was selected to perform our analyses. If the same event occurs multiple times, only the first occurrence was used for analysis.

### Statistical analysis

Continuous variables were presented as mean ± standard deviation (SD) or median (25th and 75th percentiles: P25, P75) in the case of normal or non-normal distribution, and differences between the two groups were examined by independent-sample t-test or Mann–Whitney U test correspondingly. Categorical variables were described as counts (percentages) and compared by Pearson chi-square test (Pearson χ^2^ test) or Fisher’s exact test appropriately. The Spearman’s rank correlation test or Pearson correlation test was used for evaluating the correlations between the TyG index and cardiovascular risk factors when appropriate. The Pearson correlation test was used to evaluate the correlation between two continuous variables with normal distribution, while the Spearman’s rank correlation test was applied in case that one or more of the variables being analyzed was non-normally distributed continuous variable or categorical variable. Receiver-operating characteristic (ROC) curve analysis was performed to determine the optimal cutoff point value of TyG index for predicting primary endpoint. The Kaplan–Meier survival analyses were performed to evaluate the incidence rate of adverse events between groups according to the optimal cutoff point of TyG index, and discrepancies between groups were evaluated by log-rank test. The predictive value of the variables for primary endpoint was evaluated by univariate and multivariate Cox proportional hazards analyses. The TyG index was analyzed in two ways: (1) as a categorical variable; and (2) as a continuous variable. In multivariate Cox proportional hazards analyses, four models were established to evaluate the predictive value of TyG index for primary endpoint, among which confounders were selected according to statistical significance (P < 0.2) in univariate analysis and clinical importance: (1) Model 1: adjusted for age, sex (female), BMI, SBP, DBP, smoking, drinking, duration of diabetes, dyslipidemia, prior MI, PCI, stroke and PVD; (2) Model 2: adjusted for variables included in Model 1 and diagnosis (NSTEMI), TC, HDL-C, eGFR, HbA1c, LVEF; (3) Model 3: adjusted for variables included in Model 2 and SYNTAX score, left main artery (LM) treatment, drug-coated balloon (DCB) use, complete revascularization and number of stents; (4) Model 4: adjusted for variables included in Model 3 and dual antiplatelet therapy (DAPT) at discharge, DAPT interruption in 12 months, statins at discharge, statins interruption in 12 months, oral hypoglycemic agents (metformin, alpha-glucosidase inhibitor, sulfonylurea, dipeptidyl peptidase 4 inhibitor) at discharge and insulin at discharge. The prognostic impact of TyG index for each component of primary endpoint was also assessed by using model 4. FBG and TGs were not introduced into multivariate analysis since the TyG index was calculated from them. Results of Cox proportional hazards analyses were presented as hazard ratio (HR) and 95% confidence intervals (CI). Further stratified analyses according to age (≤ 65 and > 65 years), sex, BMI (≤ 28 and > 28 kg/m^2^), hypertension, initial diagnosis (UA and NSTEMI), HbA1c (≤ 7 and > 7%), LDL-C (≤ 70 and > 70 mg/dL), and pre-admission medication including statins, oral hypoglycemic agents and insulin were employed to examine the consistence of the prognostic impact of TyG index for primary endpoint. The model used in the stratified analyses consisted of all covariates used in Model 4 except for the variables that were used for stratification. The interaction of TyG index and variables used for stratification was examined by likelihood ratio tests.

C-statistics including ROC curve analysis were performed to examine the incremental effects of TyG index on the predictive potential of the baseline risk model that including traditional risk factors. DeLong’s test was used to compare the area under the curve (AUC) from each of the models. We also calculated category-free net reclassification improvement (NRI) and integrated discrimination improvement (IDI) to determine the extent to which the addition of TyG index improves the predictive power of existing baseline risk model.

Statistical tests were performed with SPSS 23.0 (SPSS Inc., Chicago, Illinois, USA), the R Programming Language (version 3.5.1) and MedCalc version 19.1 (MedCalc Software, Belgium). A two-tailed P value < 0.05 was regarded as statistically significant.

## Results

A total of 798 patients (mean age: 60.9 ± 8.3 years; 68.3% men) were finally enrolled in present study. During the 36-month follow-up period, 17 patients (2.1% of total population) were lost to follow-up. Among the 798 participants, 180 (22.6%) experienced primary endpoint events, which consisted of 14 (1.8%) all-cause death, 37 (4.6%) non-fatal MI, and 129 (16.2%) ischemia-driven revascularization.

### Baseline characteristic of study population

Baseline characteristics of the total population and groups stratified by the occurrence of primary endpoint event were presented in Table 1. TyG index was significantly higher in patients with primary endpoint event compared with those without. Patients with a primary endpoint event showed higher age and SBP, longer duration of diabetes, and higher prevalence of dyslipidemia, previous MI and PCI history. In terms of laboratory indicators, participants with endpoint event had higher levels of TGs, TC, hs-CRP, FBG and HbA1c, but lower levels of HDL-C, eGFR and LVEF. As for the angiographic findings, those with an endpoint event showed higher proportions of LM disease, multi-vessel disease and other characteristics of complex coronary artery lesion. The SYNTAX score was significantly higher in subset with adverse prognosis. Correspondingly, more LM lesions were disposed and more coronary artery stents were implanted in patients with endpoint event. Moreover, the rate of complete revascularization was significantly lower in participants with adverse prognosis.

**Table 1:** Baseline clinical characteristics of patients with and without adverse event

	Total population (n = 798)	Without event (n = 618)	With event (n = 180)	P value
Age, years	60.9 ± 8.3	60.3 ± 8.1	62.9 ± 8.6	< *0.001*
Sex, male, n (%)	545 (68.3)	430 (69.6)	115 (63.9)	0.149
BMI, kg/m^2^	26.7 ± 3.2	26.7 ± 3.2	26.6 ± 3.2	0.772
Heart rate, bpm	71.7 ± 10.2	71.5 ± 9.8	72.1 ± 11.4	0.502
SBP, mmHg	131.8 ± 17.1	130.8 ± 16.3	135.1 ± 19.5	*0.007*
DBP, mmHg	76.8 ± 10.1	76.4 ± 9.8	78.1 ± 11.1	0.052
Smoking, n (%)	417 (52.3)	332 (53.7)	85 (47.2)	0.124
Drinking, n (%)	184 (23.1)	149 (24.1)	35 (19.4)	0.191
Family history of CAD, n (%)	93 (11.7)	73 (11.8)	20 (11.1)	0.796
Duration of diabetes, years	8.2 ± 4.3	8.0 ± 4.1	9.1 ± 4.7	*0.007*
Medical history, n (%)				
Hypertension	573 (71.8)	449 (72.7)	124 (68.9)	0.323
Dyslipidemia	710 (89.0)	537 (86.9)	173 (96.1)	*0.001*
Prior MI	175 (21.9)	118 (19.1)	57 (31.7)	< *0.001*
Prior PCI	151 (18.9)	106 (17.2)	45 (25.0)	*0.018*
Prior stroke	109 (13.7)	80 (12.9)	29 (16.1)	0.276
Prior PVD	125 (15.7)	93 (15.0)	32 (17.8)	0.375
Laboratory results				
TGs, mg/dL	138.2 (97.2, 198.5)	127.6 (91.3, 174.8)	209.1 (134.9, 299.5)	< *0.001*
TC, mg/dL	157.2 ± 39.7	153.5 ± 39.4	170.2 ± 38.2	< *0.001*
LDL-C, mg/dL	94.2 ± 33.1	93.2 ± 33.9	97.6 ± 29.8	0.112
HDL-C, mg/dL	36.9 ± 8.7	37.3 ± 8.8	35.3 ± 8.0	*0.005*
hs-CRP, mg/L	1.6 (0.7, 4.1)	1.5 (0.6, 3.9)	2.0 (0.9, 4.4)	*0.011*
Creatinine, mg/dL	0.8 ± 0.2	0.8 ± 0.2	0.8 ± 0.2	0.384
eGFR, mL/(min * 1.73 m^2^)	96.5 ± 21.6	97.4 ± 21.7	93.5 ± 21.1	*0.030*
Uric acid, μmol/L	328.0 ± 75.6	328.3 ± 75.3	327.0 ± 76.6	0.842
FBG, mg/dL	127.7 (109.6, 157.0)	125.3 (108.1, 148.4)	141.5 (118.4, 173.3)	< *0.001*
HbA1c, %	7.5 ± 1.3	7.3 ± 1.2	8.0 ± 1.3	< *0.001*
TyG index	9.1 ± 0.6	9.0 ± 0.6	9.6 ± 0.7	< *0.001*
LVEF, %	64.0 ± 6.6	64.3 ± 6.3	62.7 ± 7.5	*0.010*
Initial diagnosis, n (%)				0.149
UA	650 (81.5)	510 (82.5)	140 (77.8)	
NSTEMI	148 (18.5)	108 (17.5)	40 (22.2)	
Pre-admission medication, n (%)				
ACEI	79 (9.9)	62 (10.0)	17 (9.4)	0.816
ARB	128 (16.0)	99 (16.0)	29 (16.1)	0.976
DAPT	253 (31.7)	194 (31.4)	59 (32.8)	0.725
Aspirin	427 (53.5)	325 (52.6)	102 (56.7)	0.334
Clopidogrel	264 (33.1)	203 (32.8)	61 (33.9)	0.794
β-blocker	166 (20.8)	127 (20.6)	39 (21.7)	0.745
Statins	233 (29.2)	190 (30.7)	43 (23.9)	0.075
Proton pump inhibitor	8 (1.0)	7 (1.1)	1 (0.6)	0.796
Oral hypoglycemic agents	413 (51.8)	324 (52.4)	89 (49.4)	0.481
Metformin	170 (21.3)	138 (22.3)	32 (17.8)	0.189
Alpha-glucosidase inhibitor	185 (23.2)	140 (22.7)	45 (25.0)	0.512
Sulfonylurea	126 (15.8)	102 (16.5)	24 (13.3)	0.304
Dipeptidyl peptidase 4 inhibitor	15 (1.9)	12 (1.9)	3 (1.7)	0.811
Insulin	225 (28.2)	163 (26.4)	62 (34.4)	*0.034*
Post-discharge medication, n (%)				
ACEI	234 (29.3)	177 (28.6)	57 (31.7)	0.433
ARB	384 (48.1)	294 (47.6)	90 (50.0)	0.566
DAPT	796 (99.7)	617 (99.8)	179 (99.4)	0.934
DAPT interruption in 12 months	12 (1.5)	9 (1.5)	3 (1.7)	0.838
Aspirin	797 (99.9)	617 (99.8)	180 (100.0)	0.589
Clopidogrel	797 (99.9)	618 (100.0)	179 (99.4)	0.226
β-blocker	744 (93.2)	579 (93.7)	165 (91.7)	0.342
Statins	787 (98.6)	611 (98.9)	176 (97.8)	0.459
Statins interruption in 12 months	31 (3.9)	21 (3.4)	10 (5.6)	0.187
Proton pump inhibitor	790 (99.0)	613 (99.2)	177 (98.3)	0.554
Oral hypoglycemic agents	409 (51.3)	321 (51.9)	88 (48.9)	0.471
Metformin	167 (20.9)	135 (21.8)	32 (17.8)	0.238
Alpha-glucosidase inhibitor	181 (22.7)	137 (22.2)	44 (24.4)	0.521
Sulfonylurea	123 (15.4)	99 (16.0)	24 (13.3)	0.380
Dipeptidyl peptidase 4 inhibitor	15 (1.9)	12 (1.9)	3 (1.7)	0.811
Insulin	217 (27.2)	156 (25.2)	61 (33.9)	*0.022*
Angiographic data				
LM disease, n (%)	44 (5.5)	22 (3.6)	22 (12.2)	< *0.001*
One-vessel disease, n (%)	167 (20.9)	146 (23.6)	21 (11.7)	*0.001*
Two-vessel disease, n (%)	287 (36.0)	233 (37.7)	54 (30.0)	0.058
Three-vessel disease, n (%)	344 (43.1)	239 (38.7)	105 (58.3)	< *0.001*
Chronic total occlusion, n (%)	117 (14.7)	67 (10.8)	50 (27.8)	< *0.001*
Diffuse lesion, n (%)	237 (29.7)	169 (27.3)	68 (37.8)	*0.007*
Bifurcation lesion, n (%)	186 (23.3)	124 (20.1)	62 (34.4)	< *0.001*
In-stent restenosis, n (%)	58 (7.3)	38 (6.1)	20 (11.1)	*0.024*
SYNTAX score	12.0 ± 5.5	11.1 ± 5.1	15.2 ± 6.0	< *0.001*
Procedural results				
Target vessel territory, n (%)				
LM	25 (3.1)	14 (2.3)	11 (6.1)	*0.009*
LAD	513 (64.3)	393 (63.6)	120 (66.7)	0.449
LCX	335 (42.0)	249 (40.3)	86 (47.8)	0.073
RCA	398 (49.9)	300 (48.5)	98 (54.4)	0.164
DES implantation, n (%)	785 (98.4)	608 (98.4)	177 (98.3)	0.964
DCB use, n (%)	15 (1.9)	10 (1.6)	5 (2.8)	0.313
Complete revascularization, n (%)	414 (51.9)	333 (53.9)	81 (45.0)	*0.036*
Number of stents	2.1 ± 1.3	2.0 ± 1.2	2.4 ± 1.5	*0.001*

Italic values indicate statistically significant associations

*BMI* body mass index, *SBP* systolic blood pressure, *DBP* diastolic blood pressure, *CAD* coronary artery disease, *MI* myocardial infarction, *PCI* percutaneous coronary intervention, *PVD* peripheral vascular disease, *TGs* triglycerides, *TC* total cholesterol, *LDL-C* low-density lipoprotein cholesterol, *HDL-C* high-density lipoprotein cholesterol, *hs-CRP* high-sensitivity C-reactive protein, *eGFR* estimated glomerular filtration rate, *FBG* fasting blood glucose, *HbA1c* glycosylated hemoglobin A1c, *TyG* triglyceride glucose, *LVEF* left ventricular ejection fraction, *UA* unstable angina, *NSTEMI* non-ST-segment elevation myocardial infarction, *ACEI* angiotensin converting enzyme inhibitor, *ARB* angiotensin receptor blocker, *DAPT* dual antiplatelet therapy, *LM* left main artery, *SYNTAX* synergy between PCI with taxus and cardiac surgery, *LAD* left anterior descending artery, *LCX* left circumflex artery, *RCA* right coronary artery, *DES* drug-eluting stent, *DCB* drug-coated balloon

ROC curve analysis showed that the AUC of TyG index for predicting primary endpoint was 0.745 (95% CI 0.702–0.788, P < 0.001). The TyG index of 9.18 was determined as the optimal cutoff point for predicting primary endpoint with a sensitivity of 77.2% and a specificity of 62.8%. Baseline characteristics of groups according to the optimal cutoff point of TyG index were summarized in Table 2. Compared with patients in lower TyG index group, those with higher TyG index seemed to be younger, manifest higher levels of BMI and heart rate, and higher proportion of dyslipidemia. Laboratory indexes including TGs, TC, LDL-C, hs-CRP, uric acid, FBG and HbA1c were significantly higher in patients with higher TyG index, while HDL-C levels were relatively lower. In higher TyG index group, more patients were diagnosed as NSTEMI and prescribed insulin for treatment. Participants with higher TyG index also showed higher SYNTAX score compared to those with lower TyG index.

**Table 2:** Baseline clinical characteristics of patients stratified by the optimal cutoff point of TyG index

	Total population (n = 798)	Lower TyG index (< 9.18; n = 429)	Higher TyG index (≥ 9.18; n = 369)	P value
Age, years	60.9 ± 8.3	62.1 ± 7.9	59.5 ± 8.5	< *0.001*
Sex, male, n (%)	545 (68.3)	303 (70.6)	242 (65.6)	0.127
BMI, kg/m^2^	26.7 ± 3.2	26.3 ± 3.2	27.1 ± 3.2	< *0.001*
Heart rate, bpm	71.7 ± 10.2	70.9 ± 9.6	72.5 ± 10.8	*0.028*
SBP, mmHg	131.8 ± 17.1	131.3 ± 16.8	132.2 ± 17.5	0.443
DBP, mmHg	76.8 ± 10.1	76.2 ± 10.1	77.6 ± 10.1	0.051
Smoking, n (%)	417 (52.3)	227 (52.9)	190 (51.5)	0.688
Drinking, n (%)	184 (23.1)	106 (24.7)	78 (21.1)	0.233
Family history of CAD, n (%)	93 (11.7)	44 (10.3)	49 (13.3)	0.185
Duration of diabetes, years	8.2 ± 4.3	8.3 ± 4.3	8.2 ± 4.2	0.681
Medical history, n (%)				
Hypertension	573 (71.8)	303 (70.6)	270 (73.2)	0.426
Dyslipidemia	710 (89.0)	347 (80.9)	363 (98.4)	< *0.001*
Prior MI	175 (21.9)	90 (21.0)	85 (23.0)	0.484
Prior PCI	151 (18.9)	82 (19.1)	69 (18.7)	0.881
Prior stroke	109 (13.7)	60 (14.0)	49 (13.3)	0.772
Prior PVD	125 (15.7)	72 (16.8)	53 (14.4)	0.348
Laboratory results				
TGs, mg/dL	138.2 (97.2, 198.5)	99.2 (78.4, 127.1)	204.7 (164.4, 276.0)	< *0.001*
TC, mg/dL	157.2 ± 39.7	145.9 ± 35.0	170.4 ± 40.8	< *0.001*
LDL-C, mg/dL	94.2 ± 33.1	88.2 ± 30.5	101.1 ± 34.6	< *0.001*
HDL-C, mg/dL	36.9 ± 8.7	38.7 ± 9.3	34.8 ± 7.3	< *0.001*
hs-CRP, mg/L	1.6 (0.7, 4.1)	1.3 (0.6, 4.2)	1.9 (0.9, 3.9)	*0.009*
Creatinine, mg/dL	0.8 ± 0.2	0.8 ± 0.2	0.8 ± 0.2	0.655
eGFR, mL/(min * 1.73 m^2^)	96.5 ± 21.6	96.6 ± 21.3	96.5 ± 22.0	0.992
Uric acid, μmol/L	328.0 ± 75.6	322.2 ± 74.9	334.8 ± 75.9	*0.019*
FBG, mg/dL	127.7 (109.6, 157.0)	115.4 (102.3, 133.1)	149.9 (125.3, 177.8)	< *0.001*
HbA1c, %	7.5 ± 1.3	7.1 ± 1.2	7.8 ± 1.3	< *0.001*
TyG index	9.1 ± 0.6	8.6 ± 0.4	9.7 ± 0.4	< *0.001*
LVEF, %	64.0 ± 6.6	64.0 ± 6.8	64.0 ± 6.4	0.986
Initial diagnosis, n (%)				*0.022*
UA	650 (81.5)	362 (84.4)	288 (78.0)	
NSTEMI	148 (18.5)	67 (15.6)	81 (22.0)	
Pre-admission medication, n (%)				
ACEI	79 (9.9)	44 (10.3)	35 (9.5)	0.716
ARB	128 (16.0)	66 (15.4)	62 (16.8)	0.586
DAPT	253 (31.7)	136 (31.7)	117 (31.7)	0.999
Aspirin	427 (53.5)	226 (52.7)	201 (54.5)	0.613
Clopidogrel	264 (33.1)	141 (32.9)	123 (33.3)	0.889
β-blocker	166 (20.8)	92 (21.4)	74 (20.1)	0.629
Statins	233 (29.2)	127 (29.6)	106 (28.7)	0.786
Proton pump inhibitor	8 (1.0)	4 (0.9)	4 (1.1)	0.830
Oral hypoglycemic agents	413 (51.8)	220 (51.3)	193 (52.3)	0.773
Metformin	170 (21.3)	101 (23.5)	69 (18.7)	0.096
Alpha-glucosidase inhibitor	185 (23.2)	100 (23.3)	85 (23.0)	0.927
Sulfonylurea	126 (15.8)	67 (15.6)	59 (16.0)	0.886
Dipeptidyl peptidase 4 inhibitor	15 (1.9)	7 (1.6)	8 (2.2)	0.578
Insulin	225 (28.2)	109 (25.4)	116 (31.4)	0.059
Post-discharge medication, n (%)				
ACEI	234 (29.3)	114 (26.6)	120 (32.5)	0.066
ARB	384 (48.1)	204 (47.6)	180 (48.8)	0.729
DAPT	796 (99.7)	429 (100.0)	367 (99.5)	0.214
DAPT interruption in 12 months	12 (1.5)	7 (1.6)	5 (1.4)	0.749
Aspirin	797 (99.9)	429 (100.0)	368 (99.7)	0.462
Clopidogrel	797 (99.9)	429 (100.0)	368 (99.7)	0.462
β-blocker	744 (93.2)	400 (93.2)	344 (93.2)	0.993
Statins	787 (98.6)	423 (98.6)	364 (98.6)	0.958
Statins interruption in 12 months	31 (3.9)	14 (3.3)	17 (4.6)	0.327
Proton pump inhibitor	790 (99.0)	426 (99.3)	364 (98.6)	0.568
Oral hypoglycemic agents	409 (51.3)	217 (50.6)	192 (52.0)	0.683
Metformin	167 (20.9)	98 (22.8)	69 (18.7)	0.151
Alpha-glucosidase inhibitor	181 (22.7)	97 (22.6)	84 (22.8)	0.959
Sulfonylurea	123 (15.4)	64 (14.9)	59 (16.0)	0.676
Dipeptidyl peptidase 4 inhibitor	15 (1.9)	7 (1.6)	8 (2.2)	0.578
Insulin	217 (27.2)	104 (24.2)	113 (30.6)	*0.043*
Angiographic data				
LM disease, n (%)	44 (5.5)	19 (4.4)	25 (6.8)	0.148
One-vessel disease, n (%)	167 (20.9)	92 (21.4)	75 (20.3)	0.698
Two-vessel disease, n (%)	287 (36.0)	163 (38.0)	124 (33.6)	0.197
Three-vessel disease, n (%)	344 (43.1)	174 (40.6)	170 (46.1)	0.117
Chronic total occlusion, n (%)	117 (14.7)	55 (12.8)	62 (16.8)	0.113
Diffuse lesion, n (%)	237 (29.7)	123 (28.7)	114 (30.9)	0.493
Bifurcation lesion, n (%)	186 (23.3)	93 (21.7)	93 (25.2)	0.240
In-stent restenosis, n (%)	58 (7.3)	28 (6.5)	30 (8.1)	0.384
SYNTAX score	12.0 ± 5.5	11.6 ± 5.5	12.6 ± 5.6	*0.010*
Procedural results				
Target vessel territory, n (%)				
LM	25 (3.1)	14 (3.3)	11 (3.0)	0.819
LAD	513 (64.3)	274 (63.9)	239 (64.8)	0.791
LCX	335 (42.0)	185 (43.1)	150 (40.7)	0.480
RCA	398 (49.9)	211 (49.2)	187 (50.7)	0.674
DES implantation, n (%)	785 (98.4)	425 (99.1)	360 (97.6)	0.163
DCB use, n (%)	15 (1.9)	5 (1.2)	10 (2.7)	0.109
Complete revascularization, n (%)	414 (51.9)	229 (53.4)	185 (50.1)	0.360
Number of stents	2.1 ± 1.3	2.1 ± 1.3	2.1 ± 1.3	0.700

The groups were stratified by the optimal cutoff point of TyG index determined by ROC curve analysis

Italic values indicate statistically significant associations

*BMI* body mass index, *SBP* systolic blood pressure, *DBP* diastolic blood pressure, *CAD* coronary artery disease, *MI* myocardial infarction, *PCI*percutaneous coronary intervention, *PVD* peripheral vascular disease, *TGs* triglycerides, *TC* total cholesterol, *LDL-C* low-density lipoprotein cholesterol, *HDL-C* high-density lipoprotein cholesterol, *hs-CRP* high-sensitivity C-reactive protein, *eGFR* estimated glomerular filtration rate, *FBG* fasting blood glucose, *HbA1c* glycosylated hemoglobin A1c, *TyG* triglyceride glucose, *LVEF* left ventricular ejection fraction, *UA* unstable angina, *NSTEMI* non-ST-segment elevation myocardial infarction, *ACEI* angiotensin converting enzyme inhibitor, *ARB* angiotensin receptor blocker, *DAPT* dual antiplatelet therapy, *LM* left main artery, *SYNTAX* synergy between PCI with taxus and cardiac surgery, *LAD* left anterior descending artery, *LCX* left circumflex artery, *RCA* right coronary artery, *DES* drug-eluting stent, *DCB* drug-coated balloon

### Correlation between the TyG index and cardiovascular risk factors

The Spearman’s rank or Pearson correlation analysis was performed to determine the correlation between the TyG index and traditional or commonly-used risk factors for cardiovascular disease. The TyG index was positively correlated with BMI, FBG, HbA1c, TGs, TC, LDL-C, uric acid, and hs-CRP, while negatively correlated with age and HDL-C (Table 3).

**Table 3:** Correlations between the TyG index and traditional cardiovascular risk factors

	Correlation coefficient	P value
Age	− 0.194	< *0.001*
Sex, female	0.069	0.051
BMI	0.184	< *0.001*
FBG	0.588	< *0.001*
HbA1c	0.352	< *0.001*
TGs	0.906	< *0.001*
TC	0.333	< *0.001*
LDL-C	0.197	< *0.001*
HDL-C	− 0.273	< *0.001*
Uric acid	0.093	*0.008*
eGFR	0.010	0.785
hs-CRP	0.123	*0.001*
LVEF	0.001	0.981
SYNTAX score	0.049	0.166

Italic values indicate statistically significant associations

*BMI* body mass index, *FBG* fasting blood glucose, *HbA1c* glycosylated hemoglobin A1c, *TGs* triglycerides, *TC* total cholesterol, *LDL-C* low-density lipoprotein cholesterol, *HDL-C* high-density lipoprotein cholesterol, *eGFR* estimated glomerular filtration rate, *hs-CRP* high-sensitivity C-reactive protein, *LVEF* left ventricular ejection fraction, *SYNTAX* synergy between PCI with taxus and cardiac surgery

### Clinical outcomes and Kaplan–Meier analysis

During the 36-month follow-up period, 180 (22.6%) endpoint events including 14 (1.8%) all-cause death, 37 (4.6%) non-fatal MI, and 129 (16.2%) ischemia-driven revascularization were documented to perform the present analyses. The incidence of adverse prognosis was compared between groups stratified by the optimal cutoff point of TyG index determined by ROC curve analysis. The incidence of primary endpoint, non-fatal MI and ischemia-driven revascularization increased significantly in patients with higher TyG index compared with those with lower TyG index (all chi-square P < 0.001). However, the all-cause death rate was similar between the two groups (chi-square P = 0.172) (Table 4).

**Table 4:** Incidence of endpoint events according to the optimal cutoff point of TyG index

	Lower TyG index (< 9.18; n = 429)	Higher TyG index (≥ 9.18; n = 369)	P value
Primary endpoint, n (%)	41 (9.6)	139 (37.7)	< *0.001*
All-cause death, n (%)	5 (1.2)	9 (2.4)	0.172
Non-fatal MI, n (%)	9 (2.1)	28 (7.6)	< *0.001*
Ischemia-driven revascularization, n (%)	27 (6.3)	102 (27.6)	< *0.001*

The groups were stratified by the optimal cutoff point of TyG index determined by ROC curve analysis

Italic values indicate statistically significant associations

*TyG* triglyceride glucose, *MI* myocardial infarction

**Fig. 2:**
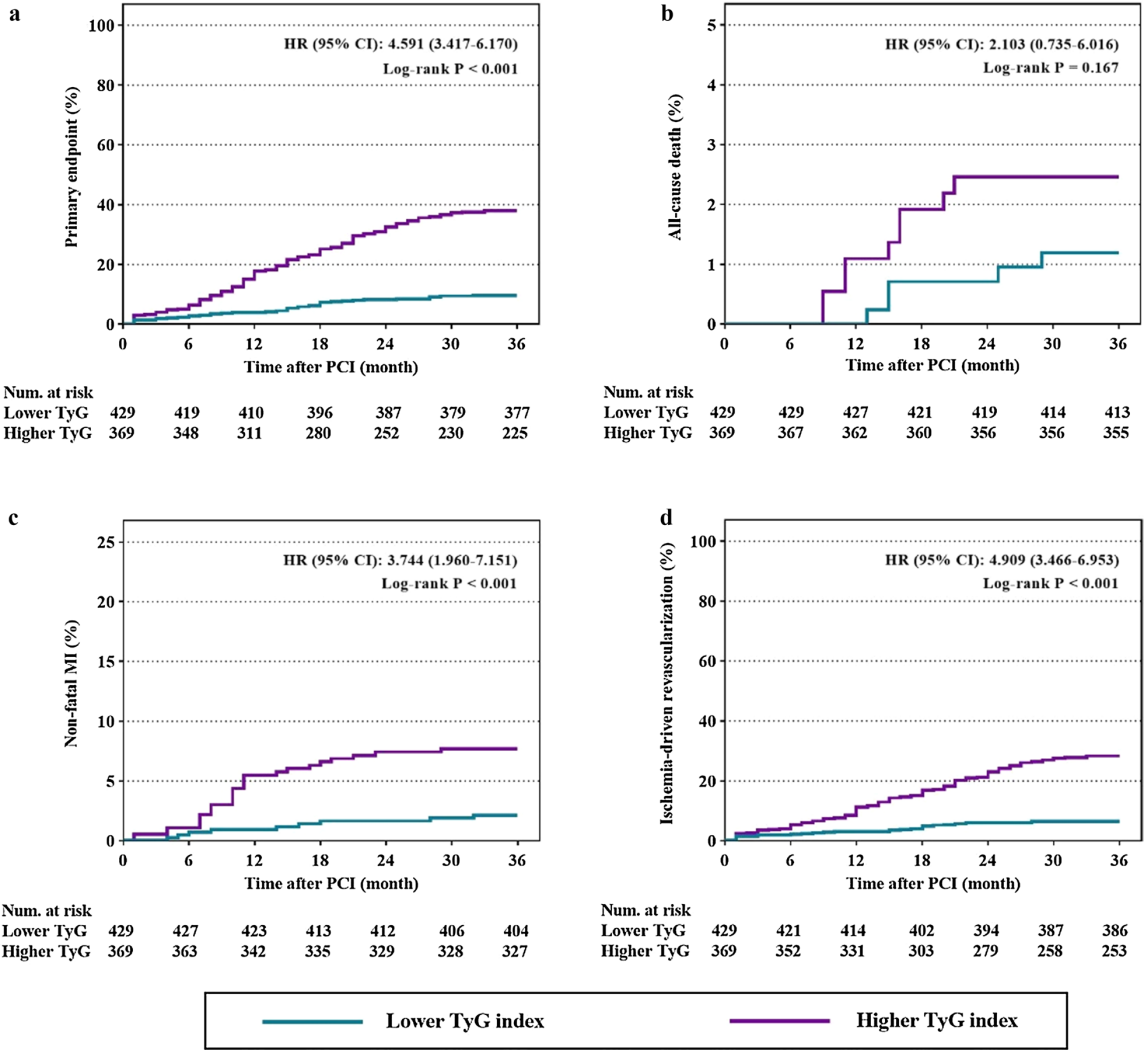
Kaplan–Meier curves for endpoint events according to the optimal cutoff point of TyG index. **a** Kaplan–Meier curves for primary endpoint; **b** Kaplan–Meier curves for all-cause death; **c** Kaplan–Meier curves for non-fatal MI; **d** Kaplan-Meier curves for ischemia-driven revascularization. The groups were stratified by the optimal cutoff point of TyG index determined by ROC curve analysis. *TyG* triglyceride glucose, *MI* myocardial infarction, *PCI* percutaneous coronary intervention, *HR* hazard ratio, *CI* confidence interval

Kaplan–Meier curves for incidence of primary endpoint and each component of it according to the optimal cutoff point of TyG index were shown in Fig. 2. Kaplan–Meier curves for primary endpoint showed a significant difference between the lower and higher TyG index group (Fig. 2a, Log-rank P < 0.001). The difference was mainly driven by the increased incidence of non-fatal MI and ischemia-driven revascularization (Fig. 2c, d, both Log-rank P < 0.001). Kaplan–Meier curves for all-cause death between the lower and higher TyG index group failed to reach statistical significance (Fig. 2b, Log-rank P = 0.167).

### Cox proportional hazard analyses to evaluate the prognostic implication of TyG index

In multivariate Cox proportional hazard analysis, four models (Model 1–4 as described above) including variables that had statistical significance (P < 0.2) and/or clinical importance were constructed to evaluate the predictive potential of TyG index for primary endpoint. After adjusting for confounding variables, higher TyG index remained to be an independent risk predictor of primary endpoint, despite of regarding TyG index as a nominal or continuous variable (all P < 0.001 in Model 1–4) (Table 5). The detailed information of Model 4 was shown in Additional file 1: Table S1.

**Table 5:** Predictive value of TyG index for primary endpoint in different Cox proportional hazards models

	TyG index as a nominal variable^a^			TyG index as a continuous variable^b^		
	HR	95% CI	P value	HR	95% CI	P value
Crude model	4.610	3.253–6.533	< *0.001*	3.367	2.677–4.235	< *0.001*
Model 1	4.858	3.367–7.011	< *0.001*	3.459	2.731–4.381	< *0.001*
Model 2	3.774	2.553–5.580	< *0.001*	2.900	2.194–3.832	< *0.001*
Model 3	3.994	2.699–5.991	< *0.001*	3.031	2.294–4.005	< *0.001*
Model 4	4.062	2.732–6.040	< *0.001*	3.208	2.400–4.289	< *0.001*

Model 1: adjusted for age, sex (female), BMI, SBP, DBP, smoking, drinking, duration of diabetes, dyslipidemia, prior MI, PCI, stroke and PVD

Model 2: adjusted for variables included in Model 1 and diagnosis (NSTEMI), TC, HDL-C, eGFR, HbA1c, LVEF

Model 3: adjusted for variables included in Model 2 and SYNTAX score, LM treatment, DCB use, complete revascularization and number of stents

Model 4: adjusted for variables included in Model 3 and DAPT at discharge, DAPT interruption in 12 months, statins at discharge, statins interruption in 12 months, oral hypoglycemic agents (metformin, alpha-glucosidase inhibitor, sulfonylurea, dipeptidyl peptidase 4 inhibitor) at discharge and insulin at discharge

Italic values indicate statistically significant associations

*TyG* triglyceride glucose, *HR* hazard ratio, *CI* confidence interval

^a^The HR was examined regarding lower TyG index as reference (stratified by the optimal cutoff point of TyG index determined by ROC curve analysis)

^b^The HR was examined by per 1-unit increase of TyG index

The predictive value of TyG index for each component of primary endpoint was also evaluated by using model 4. The results showed that a 1-unit increase of TyG index was independently associated with higher risk of non-fatal MI and ischemia-driven revascularization [HR (95% CI) for non-fatal MI: 3.332 (1.730–6.415), P < 0.001; HR (95% CI) for ischemia-driven revascularization: 3.021 (2.167–4.211), P < 0.001]. However, higher TyG index levels failed to be a predictor of all-cause death, which was consistent with the results of Kaplan–Meier curves (Table 6).

**Table 6:** Predictive value of TyG index for primary endpoint and each component in univariate and multivariate analysis

	Univariate analysis			Multivariate analysis^c^		
	HR	95% CI	P value	HR	95% CI	P value
TyG index as a nominal variable^a^						
Primary endpoint	4.610	3.253–6.533	< *0.001*	4.062	2.732–6.040	< *0.001*
All-cause death	2.103	0.705–6.276	0.183	0.872	0.179–4.258	0.866
Non-fatal MI	3.744	1.767–7.935	*0.001*	2.260	0.894–5.715	0.085
Ischemia-driven revascularization	4.920	3.218–7.521	< *0.001*	4.980	3.075–8.067	< *0.001*
TyG index as a continuous variable^b^						
Primary endpoint	3.367	2.677–4.235	< *0.001*	3.208	2.400–4.289	< *0.001*
All-cause death	1.358	0.610–3.024	0.454	0.429	0.111–1.659	0.220
Non-fatal MI	4.449	2.684–7.373	< *0.001*	3.332	1.730–6.415	< *0.001*
Ischemia-driven revascularization	2.874	2.216–3.727	< *0.001*	3.021	2.167–4.211	< *0.001*

Italic values indicate statistically significant associations

*TyG* triglyceride glucose, *MI* myocardial infarction, *HR* hazard ratio, *CI* confidence interval

^a^The HR was examined regarding lower TyG index as reference (stratified by the optimal cutoff point of TyG index determined by ROC curve analysis)

^b^The HR was examined by per 1-unit increase of TyG index

^c^The multivariate analysis was performed by using Model 4 [adjusted for age, sex (female), BMI, SBP, DBP, smoking, drinking, duration of diabetes, dyslipidemia, prior MI, PCI, stroke, PVD, diagnosis (NSTEMI), TC, HDL-C, eGFR, HbA1c, LVEF, SYNTAX score, LM treatment, DCB use, complete revascularization, number of stents, DAPT at discharge, DAPT interruption in 12 months, statins at discharge, statins interruption in 12 months, oral hypoglycemic agents (metformin, alpha-glucosidase inhibitor, sulfonylurea, dipeptidyl peptidase 4 inhibitor) at discharge and insulin at discharge]

**Fig. 3:**
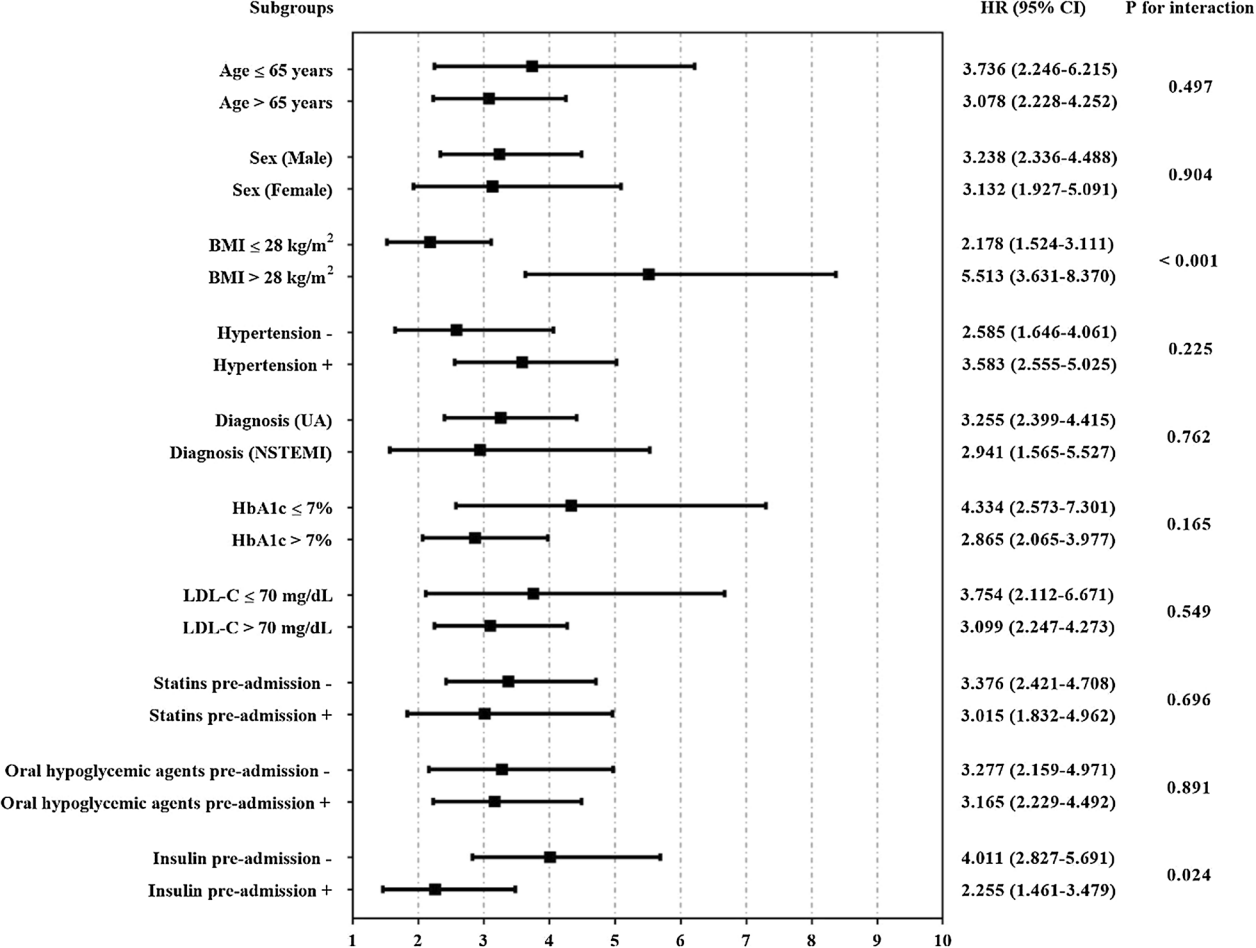
Cox proportional hazards analysis evaluating prognostic implication of TyG index in various stratifications. HR was evaluated by 1-unit increase of TyG index. *BMI* body mass index, *UA* unstable angina, *NSTEMI* non-ST-segment elevation myocardial infarction, *HbA1c* glycosylated hemoglobin A1c, *LDL-C* low-density lipoprotein cholesterol, *HR* hazard ratio, *CI* confidence interval

Further evaluation of the risk stratification value of TyG index for primary endpoint was performed in various subclasses of the study population. Increased TyG index (per 1-unit) was consistently related to primary endpoint in various subgroups, including age ≤ 65 or > 65 years, female or male, BMI ≤ 28 or > 28 kg/m^2^, with or without hypertension, UA or NSTEMI, HbA1c ≤ 7 or > 7%, LDL-C ≤ 70 or > 70 mg/dL, with or without pre-admission medication including statins, oral hypoglycemic agents and insulin (Fig. 3). Interestingly, the predictive value of TyG index seemed to be more prominent in patients with BMI > 28 kg/m^2^ [HR (95% CI) BMI > 28 kg/m^2^ 5.513 (3.631–8.370) vs. BMI ≤ 28 kg/m^2^ 2.178 (1.524–3.111), P for interaction < 0.001] and without pre-admission insulin therapy [HR (95% CI) without insulin 4.011 (2.827–5.691) vs. with insulin 2.255 (1.461–3.479), P for interaction = 0.024] (Fig. 3).

### Incremental effect of TyG index on predictive value for adverse prognosis

**Fig. 4:**
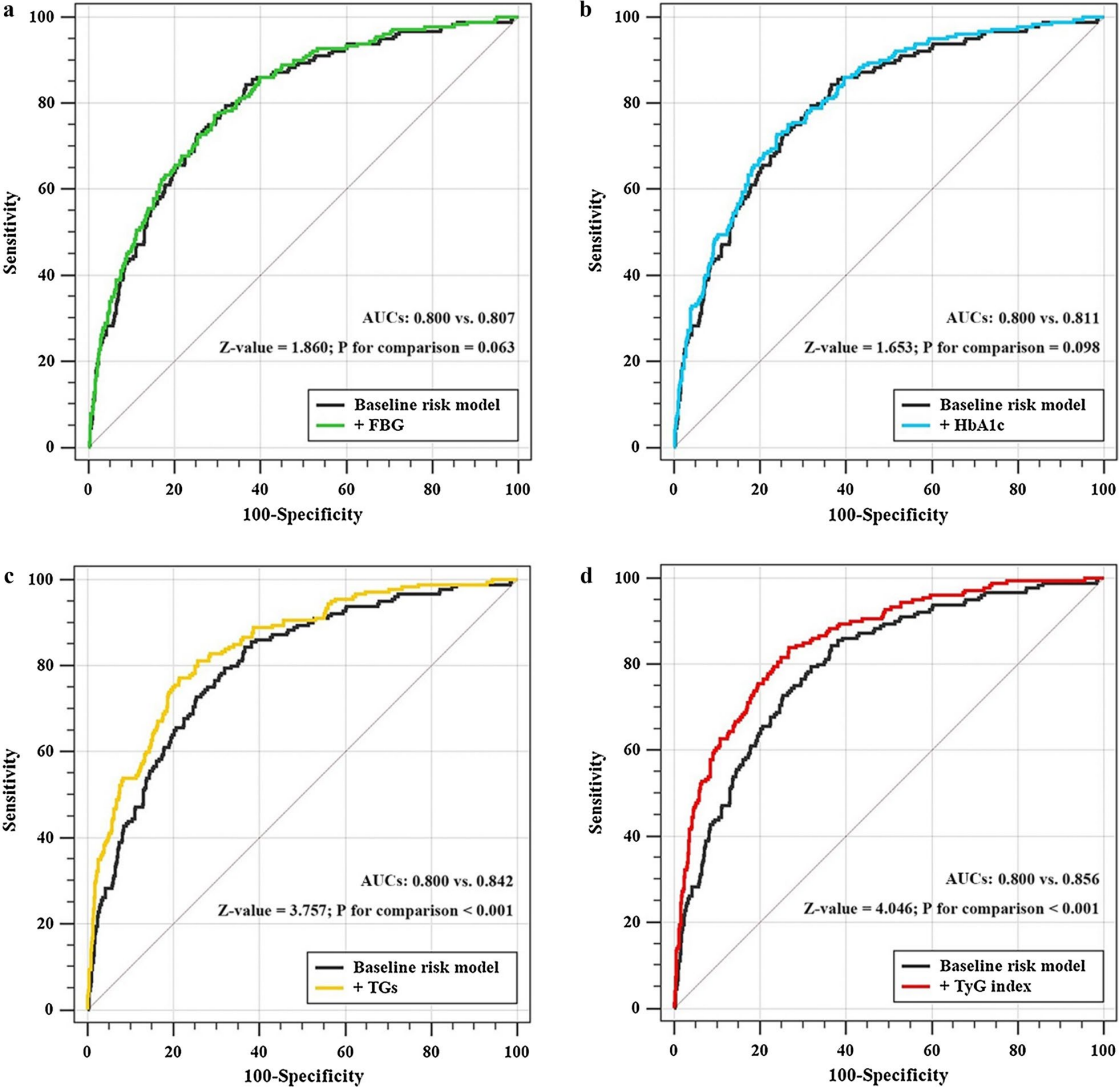
C-statistics evaluating incremental effect of FBG, HbA1c, TGs or TyG index beyond baseline risk model. **a** Baseline risk model vs. +FBG; **b** baseline risk model vs. +HbA1c; **c** baseline risk model vs. +TGs; **d** baseline risk model vs. +TyG index. The baseline risk model includes age, sex (female), smoking, SBP, prior MI, prior PCI, TC, HDL-C, eGFR, LVEF, SYNTAX score, LM treatment, complete revascularization, number of stents and statins at discharge. *FBG* fasting blood glucose, *HbA1c* glycosylated hemoglobin A1c, *TGs* triglycerides, *TyG* triglyceride glucose, *AUC* area under the curve

The addition of TyG index had a significant incremental effect on the AUC obtained from baseline risk model that consisted of risk factors including age, sex (female), smoking, SBP, prior MI, prior PCI, TC, HDL-C, eGFR, LVEF, SYNTAX score, LM treatment, complete revascularization, number of stents and statins at discharge (AUC: baseline risk model, 0.800 vs. baseline risk model + TyG index, 0.856, P for comparison < 0.001) (Table 7, Fig. 4d). Moreover, the addition of TyG index significantly improved the reclassification and discrimination ability beyond the baseline risk model with a category-free NRI of 0.346 and an IDI of 0.087 (both P < 0.001) (Table 8). Adding TGs to the baseline risk model also had a significant incremental effect on prognostic prediction (AUC: baseline risk model, 0.800 vs. baseline risk model + TGs, 0.842, P for comparison < 0.001; category-free NRI: 0.318, P < 0.001; IDI: 0.067, P < 0.001) (Tables 7 and 8, Fig. 4c). However, the addition of glycemic index including FBG or HbA1c did not have a significant incremental effect on the AUC of the baseline risk model (Table 7, Fig. 4a, b). A significant but relatively minor incremental effect on the reclassification and discrimination ability was found after adding HbA1c to the baseline risk model (Table 8).

**Table 7:** C-statistics for discrimination ability of various models

	AUC	95% CI	P value	Z value	P for comparison
Baseline risk model^a^	0.800	0.771–0.827	< *0.001*	Reference	Reference
+ FBG	0.807	0.778–0.834	< *0.001*	1.860	0.063
+ HbA1c	0.811	0.782–0.838	< *0.001*	1.653	0.098
+ TGs	0.842	0.815–0.867	< *0.001*	3.757	< *0.001*
+ TyG index	0.856	0.829–0.879	< *0.001*	4.046	< *0.001*

Italic values indicate statistically significant associations

*FBG* fasting blood glucose, *HbA1c* glycosylated hemoglobin A1c, *TGs* triglycerides, *TyG* triglyceride glucose, *AUC* area under the curve, *CI* confidence interval

^a^The baseline risk model includes age, sex (female), smoking, SBP, prior MI, prior PCI, TC, HDL-C, eGFR, LVEF, SYNTAX score, LM treatment, complete revascularization, number of stents and statins at discharge

**Table 8:** Category-free NRI and IDI for the incremental predictive values of various models

	Category-free NRI			IDI		
	Index	95% CI	P value	Index	95% CI	P value
Baseline risk model^a^	–	–	Reference	–	–	Reference
+ FBG	0.076	− 0.146 to 0.190	0.358	0.005	− 0.005 to 0.019	0.408
+ HbA1c	0.145	0.050–0.234	*0.020*	0.014	0.001–0.038	*0.020*
+ TGs	0.318	0.143–0.399	< *0.001*	0.067	0.028–0.108	< *0.001*
+ TyG index	0.346	0.230–0.430	< *0.001*	0.087	0.039–0.128	< *0.001*

*FBG* fasting blood glucose, *HbA1c* glycosylated hemoglobin A1c, *TGs* triglycerides, *TyG* triglyceride glucose, *NRI* net reclassification improvement, *IDI* integrated discrimination improvement, *CI* confidence interval

^a^The baseline risk model includes age, sex (female), smoking, SBP, prior MI, prior PCI, TC, HDL-C, eGFR, LVEF, SYNTAX score, LM treatment, complete revascularization, number of stents and statins at discharge

## Discussion

In our present study, we retrospectively investigated the predictive significance of IR assessed by TyG index for adverse prognosis in patients with T2DM and NSTE-ACS who were treated with PCI. The major findings are listed as follows: (1) the TyG index was significantly correlated with variety of risk factors for cardiovascular disease; (2) compared to participants with lower TyG index, those with higher TyG index had an apparently higher incidence of primary endpoint; (3) the increased level of TyG index was a strong indicator of worse prognosis in the study population, even after adjustment of confounding risk factors; (4) the addition of TyG index to the baseline risk model including traditional risk factors significantly promoted the ability of risk stratification.

T2DM has been widely recognized as the most significant risk factors for cardiovascular disease and it is very common for patients with ACS combined with T2DM. Certain studies have demonstrated that T2DM is significantly associated with preclinical cardiovascular organ damage, development of CAD, more complex coronary lesions and adverse prognosis [4–6, 29] and the association has been shown to be mediated primarily by IR [30]. It has been proved that IR is significantly related to the development and progression of coronary atherosclerosis [7, 8]. Therefore, for patients with or at high risk of CAD, quantitative assessment of the extent of IR is of great clinical importance for risk stratification and prognosis prediction. The euglycemic-hyperinsulinemic clamp has been acknowledged as the gold standard method for the diagnosis of IR by previous studies [31]. However, this method is relatively time-consuming, expensive and complicated to operate, which makes it comparatively difficult to be applied in real-world clinical practice. Homeostasis model assessment of IR (HOMA-IR), which is calculated by fasting insulin and glucose, has been one of the commonly used method for the assessment of IR in current clinical applications [31]. However, the insulin concentration is not routinely measured in clinical practice, which makes HOMA-IR inappropriate for extensive clinical application. Based on these, a surrogate marker of IR named TyG index derived from commonly used clinical indicators (fasting TGs and glucose) has been proposed and showed to be well related to the euglycemic-hyperinsulinemic clamp and HOMA-IR [11, 32–34]. And studies even showed that the TyG index may have a better performance on the prediction of IR and atherosclerosis compared with HOMA-IR [35, 36].

Previous studies have demonstrated that IR evaluated by TyG index is strongly related to the incidence of diabetes and prediabetic status, suggesting that TyG index may be a considerable predictor for early identifying individuals at high risk of developing diabetes and prediabetes, even performs better than other risk factors such as FBG and weight gain [14–16, 37, 38]. Studies also showed that elevated level of TyG index is prominently associated with an increased risk of developing cardiovascular disease including CAD and ischemic stroke, which suggests evaluation of TyG index might be helpful for identifying people who is susceptible to cardiovascular disease, despite existence of traditional cardiovascular risk factors or not [17, 18, 39–41]. And for patients with stable CAD, TyG index has been demonstrated to be positively related to future adverse clinical outcomes, indicating that TyG index may play an important role in the prediction of clinical prognosis in patients with stable CAD [42, 43]. The clinical significance of TyG index has been increasing as the adverse effects of it on individuals with or at high risk of cardiovascular disease have been elucidated. Evaluation of TyG index may have great clinical importance on risk stratification and therapeutic individuation for these patients.

Several studies have shown that there is an important correlation between TyG index and clinical prognosis in patients with ACS. Study from Mao et al. [44] revealed that the level of TyG index is strongly associated with the complexity of coronary lesions and the incidence of future adverse cardiovascular event during a 12-month of follow-up in patients diagnosed with NSTE-ACS. Another observational study from Luo et al. [45] assessing the predictive potential of TyG index for 1-year prognosis suggested that the increased TyG index might be an effective indicator of worse prognosis in patients with ST-segment elevation myocardial infarction (STEMI) who were treated with PCI. However, whether the predictive value of TyG index for poor prognosis was consistent in patients with or without diabetes was not investigated in former studies. Ma et al. [46] evaluated the predictive significance of TyG index in participants with T2DM and ACS undergoing PCI and showed that the TyG index was the independent predictor of adverse clinical outcomes. However, whether the addition of TyG index has an incremental effect on predicting adverse cardiovascular prognosis at the basis of traditional risk factors is not confirmed. The present study, which has a relatively longer follow-up period, revealed the significant prognostic impact of TyG index and its incremental effect on risk stratification at the basis of traditional risk factors in a specific cohort of patients with T2DM and NSTE-ACS undergoing PCI, which makes the study be great agreement and complement to previous literatures.

Based on the formula used for calculating TyG index, it’s easy to conclude that the value of TyG index is determined by the levels of fasting TGs and FPG. Therefore, factors manipulating these two indicators such as statins, TGs-lowering therapies and antidiabetic medications all have certain impacts on the evaluation of TyG index [47, 48]. The results of current study showed that the addition of TyG index or fasting TGs, but not FBG, had a significant incremental effect on predictive performance at the basis of a baseline risk model. This may be mainly attributed to the large proportion of participants receiving antidiabetic medications before admission, which can influence the assessment of the true level of FBG, thus further affecting its predictive value for adverse prognosis. The exclusion of patients receiving TGs-lowering therapies mitigated the influence of TGs-lowering medications on fasting TGs levels to great extent, so the fasting TGs levels manifested a significant discriminative performance of predicting adverse prognosis beyond a baseline risk model including traditional risk factors. The stratification analysis according to receiving statins, oral hypoglycemic agents and insulin or not showed that the predictive value of TyG index was more significant in patients without insulin treatment before admission [HR (95% CI) without insulin 4.011 (2.827–5.691) vs. with insulin 2.255 (1.461–3.479), P for interaction = 0.024], which indicates that the antidiabetic therapies, especially insulin, do have an important effect on predictive performance of TyG index for adverse prognosis.

The potential mechanism inducing the association of IR presented by TyG index with development and progression of cardiovascular disease remains uncertain, several speculations summarize as follows. (1) It has been demonstrated that TyG index is closely related to traditional risk factors for cardiovascular disease such as hypertension [49] and renal insufficiency [50]. In the present study, participants with higher TyG index exactly tended to combine with more severe and complex clinical conditions in terms of BMI, blood pressure, lipid profiles and coronary lesions, and correlation analysis also showed that TyG index is positively related to multiple risk factors for cardiovascular disease. (2) Study have shown that FBG mainly reflects IR from liver, whereas fasting TGs mainly reflects IR from adipose cell [51]. Therefore, it can be concluded that TyG index may reflect IR from two aspects and thus be closely related to IR, which has been widely demonstrated to have significant relationship with endothelial dysfunction, oxidative stress, cardio-vascular remodeling, coagulation imbalance and inflammation response [52–54]. Indeed, a positive association between TyG index and hs-CRP levels was confirmed in the present study. (3) Certain studies have also identified an important correlation between TyG index and coronary artery calcification [55], which may be another potential mechanism. (4) The TyG index has been also demonstrated to be related to arterial stiffness evaluated by pulse pressure, brachial-ankle pulse wave velocity and carotid-femoral pulse wave velocity, which has been recognized as cardiovascular risk predictor [10, 56–58].

Since adverse prognostic impacts of IR on individuals with CAD have been elucidated by previous studies, taking assessment and intervention of IR into long-term management strategies may be beneficial for patients with CAD. However, the relative lack of research about intervention on IR in patients with CAD makes it uncertain whether intervention of IR is necessary for the management of such patients. Former studies have shown that whole-grain consumption plays a significant protective role on IR and inflammatory markers [59, 60]. However, a recent systematic review of 9 RCTs indicated that there is insufficient evidence on the effect of whole-grain diets on cardiovascular outcomes or major cardiovascular disease risk factors [61]. This may be partly attributed to the fact that the association between whole-grain consumption and IR is partially mediated by adiposity [60]. Our present study also revealed that the predictive value of IR presented by TyG index seemed to be more prominent in patients BMI > 28 kg/m^2^ [HR (95% CI) BMI > 28 kg/m^2^ 4.625 (2.863–7.471) vs. BMI ≤ 28 kg/m^2^ 2.355 (1.749–3.170), P for interaction = 0.044]. Further specific-designed studies are required to determine whether interventions of IR assessed by TyG index have a positive impact on improving clinical prognosis in this population.

This study confirmed the predictive value of IR presented by TyG index for adverse prognosis in a cohort including patients with T2DM and NSTE-ACS who were treated with PCI, which indicates that TyG index can be an available predictor in clinical practice and has a positive effect on more comprehensive risk evaluation and stratification on the basis of traditional risk factors in this selected population. Meanwhile, some limitations of the study should be recognized. (1) This study is a single-center, retrospective, observational study in a highly selected cohort with strict exclusion criteria, and the sample size is relatively small, which may weaken the power of the results. Further prospective, multi-center study in a more extensive population with larger sample size are needed to further verify the present findings. (2) The TyG index was assessed only once at admission. The changes of TyG index during the follow-up period, which may have better prediction value for adverse prognosis, were not assessed in our study. (3) Certain proportion of participants received statins therapy and antidiabetic treatment at admission, which may have potential impact on the TyG index and the study results. (4) Non-fatal stroke and cardiovascular death, the commonly-used endpoint events, were not specified in current study since the information about them was relatively inadequate. (5) Nearly all of the study population is Chinese patients. The results should be cautiously interpreted and expanded to Western population as differences in metabolic levels exist among different races. (6) It is hard to rule out that some patients may be complicated with undiagnosed systemic diseases, such as occult malignancies, which may have impact on the assessment of prognosis. (7) The HOMA-IR was not calculated in the present study, so the comparison between TyG index and HOMA-IR is lacking.

## Conclusions

Increased IR extent presented by TyG index is a prominent risk predictor of adverse prognosis in patients with T2DM and NSTE-ACS who were treated with PCI. The addition of the TyG index to a baseline risk model has a strong incremental effect on the predictive potential for adverse prognosis. Further prospective, randomized studies need to be performed to determine whether interventions for IR have a positive impact on improving clinical prognosis.

## Supplementary information

**Additional file 1: Table S1.** Univariate and multivariate Cox proportional hazard analysis (model 4) for primary endpoint.

## Data Availability

The datasets used and/or analyzed during the current study are available from the corresponding author on reasonable request.

## Abbreviations

CAD: Coronary artery disease
ACS: Acute coronary syndrome
T2DM: Type 2 diabetes mellitus
IR: Insulin resistance
TyG index: Triglyceride-glucose index
NSTE-ACS: Non-ST-segment elevation acute coronary syndrome
PCI: Percutaneous coronary intervention
TGs: Triglycerides
BMI: Body mass index
FBG: Fasting blood glucose
NSTEMI: Non-ST-segment elevation myocardial infarction
UA: Unstable angina
SBP: Systolic blood pressure
DBP: Diastolic blood pressure
HbA1c: Glycosylated hemoglobin A1c
TC: Total cholesterol
LDL-C: Low-density lipoprotein cholesterol
HDL-C: High-density lipoprotein cholesterol
eGFR: Estimated glomerular filtration rate
SYNTAX: The synergy between PCI with taxus and cardiac surgery
MI: Myocardial infarction
PVD: Peripheral vascular disease
LVEF: Left ventricular ejection fraction
DAPT: Dual antiplatelet therapy
ROC: Receiver operating characteristics
hs-CRP: High-sensitivity C-reactive protein
LM: Left main artery
AUC: Area under the curve
HR: Hazard ratio
CI: Confidence interval
NRI: Net reclassification improvement
IDI: Integrated discrimination improvement
HOMA-IR: Homeostasis model assessment of insulin resistance
