# Supplementary material for: Impacts of triglyceride-glucose index on prognosis of patients with type 2 diabetes mellitus and non-ST-segment elevation acute coronary syndrome: results from an observational cohort study in China
Source: Cardiovasc Diabetol. 2020 Jul 8;19:108. doi: 10.1186/s12933-020-01086-5 (PMC7341665; doi:10.1186/s12933-020-01086-5)
Supplement: Supplementary file 1 — Additional file 1: Table S1. Univariate and multivariate Cox proportional hazard analysis (model 4) for primary endpoint. [file 12933_2020_1086_MOESM1_ESM.docx]

Table S1. Univariate and multivariate Cox proportional hazard analysis (model 4) for primary endpoint.

|  | Univariate analysis | | | Multivariate analysis | | |
| --- | --- | --- | --- | --- | --- | --- |
|  | HR | 95% CI | P value | HR | 95% CI | P value |
| Age, per 10 years | 1.417 | 1.177-1.705 | **< 0.001** | 1.705 | 1.339-2.172 | **< 0.001** |
| Sex, female | 1.269 | 0.936-1.720 | 0.124 | 1.009 | 0.685-1.487 | 0.963 |
| BMI, per 1 kg/m^2^ | 0.994 | 0.949-1.041 | 0.793 | 0.984 | 0.935-1.036 | 0.535 |
| SBP, per 10 mmHg | 1.141 | 1.052-1.238 | **0.001** | 1.084 | 0.967-1.216 | 0.167 |
| DBP, per 10 mmHg | 1.150 | 1.000-1.322 | 0.050 | 1.037 | 0.856-1.256 | 0.710 |
| Smoking history | 0.797 | 0.595-1.069 | 0.130 | 0.885 | 0.603-1.299 | 0.532 |
| Drinking history | 0.780 | 0.539-1.129 | 0.188 | 1.031 | 0.663-1.601 | 0.893 |
| Duration of diabetes, per 1 years | 1.051 | 1.017-1.086 | **0.003** | 0.995 | 0.957-1.035 | 0.810 |
| Dyslipidemia | 3.227 | 1.516-6.870 | **0.002** | 1.568 | 0.687-3.579 | 0.286 |
| Prior MI | 1.819 | 1.329-2.491 | **< 0.001** | 1.723 | 1.169-2.540 | **0.006** |
| Prior PCI | 1.514 | 1.081-2.122 | **0.016** | 1.690 | 1.146-2.492 | **0.008** |
| Prior stroke | 1.271 | 0.854-1.892 | 0.236 | 0.622 | 0.200-1.932 | 0.412 |
| Prior PVD | 1.206 | 0.823-1.767 | 0.336 | 2.079 | 0.703-6.150 | 0.186 |
| Diagnosis, NSTEMI | 1.336 | 0.940-1.899 | 0.106 | 0.736 | 0.480-1.128 | 0.160 |
| TC, per 10 mg/dL | 1.079 | 1.047-1.112 | **< 0.001** | 1.030 | 0.987-1.076 | 0.176 |
| HDL-C, per 10 mg/dL | 0.780 | 0.650-0.938 | **0.008** | 0.931 | 0.727-1.191 | 0.569 |
| eGFR, per 10 mL/(min*1.73m^2^) | 0.925 | 0.863-0.992 | **0.028** | 0.971 | 0.897-1.052 | 0.470 |
| HbA1c, per 1 % | 1.355 | 1.228-1.494 | **< 0.001** | 1.070 | 0.936-1.224 | 0.320 |
| TyG index, per 1-unit | 3.367 | 2.677-4.235 | **< 0.001** | 3.208 | 2.400-4.289 | **< 0.001** |
| LVEF, per 10 % | 0.720 | 0.583-0.890 | **0.002** | 0.991 | 0.967-1.015 | 0.464 |
| DAPT at discharge | 0.437 | 0.061-3.123 | 0.410 | 0.621 | 0.050-7.693 | 0.711 |
| DAPT interruption in 12 months | 1.138 | 0.364-3.564 | 0.824 | 1.404 | 0.336-5.869 | 0.642 |
| Statins at discharge | 0.493 | 0.183-1.330 | 0.163 | 0.373 | 0.098-1.417 | 0.148 |
| Statins interruption in 12 months | 1.573 | 0.831-2.976 | 0.164 | 1.402 | 0.609-3.227 | 0.427 |
| Metformin | 0.803 | 0.548-1.177 | 0.262 | 0.829 | 0.543-1.264 | 0.383 |
| Alpha-glucosidase inhibitor | 1.109 | 0.789-1.558 | 0.551 | 0.792 | 0.548-1.143 | 0.213 |
| Sulfonylurea | 0.795 | 0.517-1.221 | 0.295 | 0.619 | 0.380-1.008 | 0.054 |
| Dipeptidyl peptidase 4 inhibitor | 0.893 | 0.285-2.797 | 0.847 | 1.048 | 0.316-3.475 | 0.939 |
| Insulin at discharge | 1.440 | 1.058-1.961 | **0.021** | 0.935 | 0.663-1.318 | 0.701 |
| SYNTAX score, per 1-unit | 1.116 | 1.089-1.143 | **< 0.001** | 1.090 | 1.058-1.123 | **< 0.001** |
| LM treatment | 2.343 | 1.273-4.313 | **0.006** | 1.333 | 0.660-2.694 | 0.423 |
| DCB use | 2.087 | 0.858-5.078 | 0.105 | 2.416 | 0.914-6.389 | 0.075 |
| Complete revascularization | 0.717 | 0.534-0.961 | **0.026** | 0.852 | 0.572-1.269 | 0.431 |
| Number of stents, per 1 stent | 1.180 | 1.064-1.308 | **0.002** | 1.154 | 1.004-1.327 | **0.044** |

BMI, body mass index; SBP, systolic blood pressure; DBP, diastolic blood pressure; MI, myocardial infarction; PCI, percutaneous coronary intervention; PVD, peripheral vascular disease; NSTEMI, non-ST-segment elevation myocardial infarction; TC, total cholesterol; HDL-C, high-density lipoprotein cholesterol; eGFR, estimated glomerular filtration rate; HbA1c, glycosylated hemoglobin A1c; TyG, triglyceride glucose; LVEF, left ventricular ejection fraction; DAPT, dual antiplatelet therapy; SYNTAX, synergy between PCI with taxus and cardiac surgery; LM, left main artery; DCB, drug-coated balloon; HR, hazard ratio; CI, confidence interval.
